# A novel parametric approach to mine gene regulatory relationship from microarray datasets

**DOI:** 10.1186/1471-2105-11-S11-S15

**Published:** 2010-12-14

**Authors:** Wanlin Liu, Dong Li, Qijun Liu, Yunping Zhu, Fuchu He

**Affiliations:** 1 State Key Laboratory of Proteomics, Beijing Proteome Research Center, Beijing Institute of Radiation Medicine, Beijing 102206, China; 2 Department of Chemistry and Biology, College of Science, National University of Defense Technology, Changsha 410073, China

## Abstract

**Background:**

Microarray has been widely used to measure the gene expression level on the genome scale in the current decade. Many algorithms have been developed to reconstruct gene regulatory networks based on microarray data. Unfortunately, most of these models and algorithms focus on global properties of the expression of genes in regulatory networks. And few of them are able to offer intuitive parameters. We wonder whether some simple but basic characteristics of microarray datasets can be found to identify the potential gene regulatory relationship.

**Results:**

Based on expression correlation, expression level variation and vectors derived from microarray expression levels, we first introduced several novel parameters to measure the characters of regulating gene pairs. Subsequently, we used the naïve Bayesian network to integrate these features as well as the functional co-annotation between transcription factors and their target genes. Then, based on the character of time-delay from the expression profile, we were able to predict the existence and direction of the regulatory relationship respectively.

**Conclusions:**

Several novel parameters have been proposed and integrated to identify the regulatory relationship. This new model is proved to be of higher efficacy than that of individual features. It is believed that our parametric approach can serve as a fast approach for regulatory relationship mining.

## Background

Gene regulation, a basic process of organisms, is important for systems biology research. Gene regulatory relationship mining can help identify the complicated regulatory networks, uncover the regulatory patterns in the cell, and expand the systematic view of biological processes.

In the past decade, as a novel high-throughput method, microarray has been widely used in genome wide research. Therefore, many algorithms have also been introduced in this field to construct gene regulatory networks based on microarray data.

A basic hypothesis among these approaches is that the variation of expression levels of transcription factors (TF) will affect expression levels of its target genes (TGs) through the regulatory relationships. In other words, the expression profiles of TF and its TGs are somewhat interrelated. Consequently, measuring the correlation of the expression profiles represented by microarrays, especially time-series microarrays, has become a natural consideration.

Some of the previous work has contributed to this task [1]. According to the characters of the models, the algorithms can be broadly classified into several different categories.

First, clustering algorithms are basic and simple methods, based on the similarity of the expression levels of TF and its TGs [2,3]. Meanwhile, some graph models are used, such as classical graphical Gaussian models [4], and the coexpression graph-based approach [5]. Secondly, a series of network models has been widely used, such as Boolean network [6-8], naïve Bayesian network [9], and dynamic Bayesian network [10-12]. These methods are considered to be the mainstream gene regulatory constructing methods. Besides, ODE (ordinary differential equation) [13], NDE (nonlinear differential equation) [14] and pDE (partial differential equations) have also been introduced, which can adjust the parameters of differential equations manually to the biological process. The mutual information method is gaining popularity [15,16] and is used to measure the entropy of the whole system.

However, most of these complex models and algorithms focus on global properties, and some of them could not offer parameters. This leaves us wondering whether some simple but potentially probable basic characteristics could be uncovered.

As the first step, we tested the basic Pearson correlation coefficient, PCC [17] to outline the relationship between gene regulatory relationships and the expression level represented by microarray. Generally, the existence of regulatory relationship is more likely while the absolute value of the PCC is larger. There are, however, some exceptions (see Additional file 1). In order to handle this problem, several indicators or parameters should be introduced. During correlation analysis, we found it necessary to take into account the variation of expression levels. Therefore, we measured the variation characters using both expression level differences (ELD) and differences of average - standard deviation (Δmean-Δδ) parameter groups. These parameters, when combined with PCC, can effectively improve the accuracy of prediction.

Moreover, we considered expression level vectors mapped on TF-TG expression level space. Given the property of time-series gene expression relationships, it is naturally assumed that different expression relationships might relate to the vectors in different quadrants. Therefore we calculated the sum vectors in different quadrants respectively and chose the representative vector as the main vector of one TF-TG pair. Afterwards, the modulus - argument (|x|-θ) parameter group was fixed.

**Figure 1:**
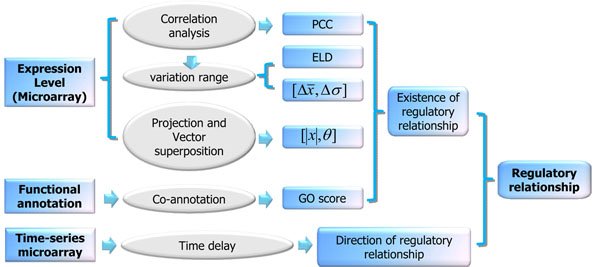
**Flowchart of our approach.** We first constructed several features to measure the gene pairs by gene expression data, assessed their reliability by likelihood ratio and finally integrated them with a naïve Bayesian model. Subsequently, the direction of the regulatory relationship has been predicted as time delay by time-series microarray.

In addition, we analyzed functional co-annotation of transcription factors and target genes before selecting the GO score as another parameter. The parameters will be integrated to perform a better prediction. Finally, we selected Bayesian models combined with joint likelihood ratio to integrate all the parameters and achieved a better performance (See Figure 1).

## Results

### Construction of multiple features to mine regulatory relationships

#### Variation of the expression levels

To deal with exceptions during the measurement by Pearson correlation coefficient, other elements besides PCC should be considered. Taking into account the levels of expression strength, we supposed that the similarity may relate to the distribution range of the variation of expression levels. e.g., the smaller the variation range of the expression levels of tested TF-TG is, the more the pair is likely to be strongly correlated (See Additional file 1).

**Figure 2:**
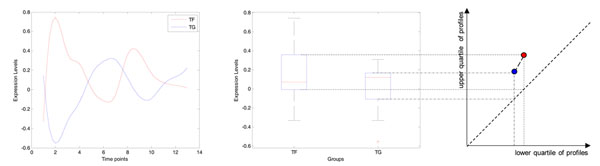
**A mapping of the expression levels onto the variation space.** The upper quartile of the expression levels of both TF and TG mapped onto the y-axis, as well as the lower quartile mapped onto the x-axis. And the Euclidean distance between the two mapping points was named the distance of expression levels’ differences.

During the analysis of the practical yeast microarray data, we recognized the necessity to take into account the dynamic range of expression levels. In order to utilize the character of time-series profiles, we measured the variation of the expression levels (series of data points). We defined a mapping of the expression levels onto the variation space (Figure 2) before we calculated the Euclidean distances between the mapped points of the original expression levels. We named the calculated Euclidean distances as the distance of expression levels’ differences (ELD). This criterion can effectively reduce false negative prediction based on PCC only.

To further study the statistic feature of expression levels, the standard deviation and their mean value were both used. The differences in the mean value and the standard deviation between TF and its TG were calculated, as in the case of another parameter group Δmean-Δδ.

#### Modulus and angle of expression level vector by vector analysis

Vector analysis was used to compare gene expression responses between different experimental backgrounds[18] according to a simple principle. The change of the gene expression against the two experimental backgrounds is represented by a vector. Both up- or down- regulation and the regulatory intensity can be showed. The various sectors of the Cartesian plane will correspond to various prototypical behaviours of genes.

Here we considered an extension of vector analysis, mapping expression levels under each condition or time point in the coordination system of TF-TG’s expression levels. Moreover, we attempted to infer the expression patterns such as correlation or inversion with or without time-shift.

**Figure 3:**
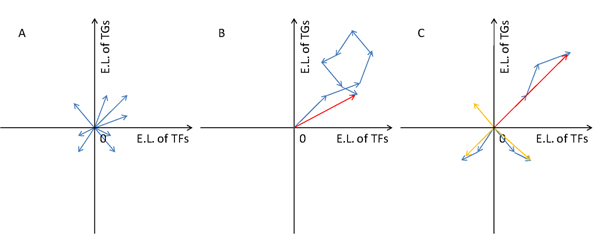
**Illustration of vector analysis** A) Mapping expression levels as sub-vectors (blue arrows) under each measurement in the coordination system of TF-TG’s expression levels. B) Sum vector (red arrow) of sub-vectors. C) Quadrantal sum vector calculation. Measurements of expression relationships corresponding to the sub-vectors in different quadrants represent different patterns. Therefore, the sum vectors (orange arrows) were calculated in different quadrants respectively, and then we chose the vector with the largest modulus as the main representative vector (red arrow) of one TF-TG pair. E.L: expression levels

Given the property of time-series gene expression relationships, it can be naturally assumed that different expression relationships might correspond to the vectors in different quadrants. So we calculated the sum vectors in different quadrants respectively before choosing the vector which has the largest modulus as the main representative vector of one TF-TG pair (Figure 3). The representative features can be grasped and amplified by vector analysis.

#### GO coannotation score

It is known that the regulatory relationship emerges in the same biological process. Here we used the Gene Ontology (GO) classification system to define the measurement of functional similarity. The GO annotation term has several hierarchical grades, and coannotation terms can be organized as a tree so that all the leaves of the tree represent the coannotation strength. A leaf with deeper (more detailed) annotation denotes stronger coannotation strength and will be assigned a higher score according to the grade of the GO term. Besides, the divergence of branches of leaves has to be considered. For example, two seven-grade coannotation terms are derived from the same five-grade node, and another two seven-grade coannotation terms do not have any common parent node. Counting the scores of the pairs according to the grade of their leaves alone will obtain the same but an obviously unfair result. So the duplicated count produced by the same parent term should be excluded. That is,

If more than two leaves are derived from one divergent node, a weight score will be subtracted.

#### Time-delay character for predicting of the direction of regulatory relationships

During the transmission of expression perturbation from the TF to TG, a time delay might occur. Therefore, a suitable parameter that can describe the existence of the time delay might help to fix the direction of the regulatory relationship.

The point which has the extremum amplitude is a character that is to be considered. If the expression profile can be regarded as the continuous function of time, i.e., *y* = *f*(*t*), the extremum exists at *y*’ = Δ*y* = d*y*/d*t* = 0. For discrete points such as the microarray time-series expression profile, this problem can be solved by difference, with the popularization of the differentiation. Boundary condition Δ*y* = 0 is often not available, so condition Δ*y*(*t*)·Δ*y*(*t* - 1) < 0 might be more appropriate.

**Figure 4:**
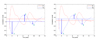
Distribution of different peaks based on mean and median values.

Besides, the basis of amplitude must be considered. Both the mean and median value might be available (Figure 4).

### Multiple features can be used to predict regulatory relations

#### Variation of expression levels

**Figure 5:**
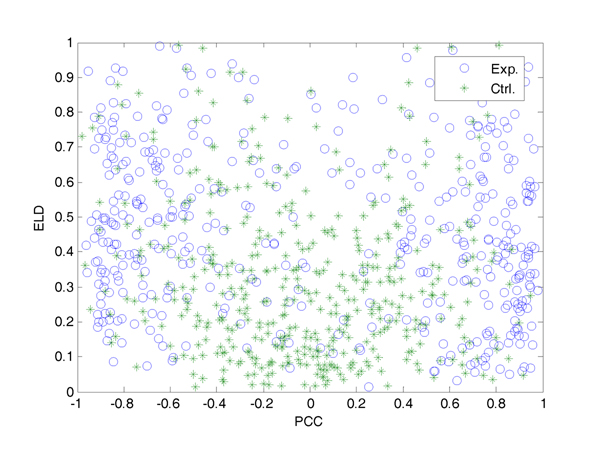
**Distribution of regulation pairs of yeast cell cycle based on PCC and ELD.** Non-existing pairs tend to cluster around the origin. Therefore, existing pairs with smaller |PCC| but relatively larger ELD avoid classification into the non-existing group, which is quite natural if based on PCC alone.

By taking into account the ELD combined with widely-used PCC, the distribution of existing and non-existing regulatory relationship pairs can be classified more distinctly (Figure 5).

As shown in Figure 5, a significant classification could be found. The green points represent the non-existing pairs relatively concentrated in the region while the blue ones represent the existing regulatory pairs in this region. Judging by PCC alone without considering the parameter ELD would lead to more false negative judgments. Now the typical non-ideal cases described in the Additional file 1 C and D might refuse to be classified falsely by PCC alone.

Furthermore, differences in standard deviation and mean value have a widely accepted definition and the accuracy of this parameter group is acceptable. So we also take the parameters Δmean - Δδ as indicators. Generally, Δmean – Δδ correspond to the likelihood ratio distribution represented by PCC. The accuracy of this parameter group is as high as that of ELD verified by J48 classification tree. However, it also has some *cons*, i.e. the parameters are pairwise, and these two parameters should be used at the same time.

#### Modulus and angle of expression levels vector by vector analysis

**Figure 6:**
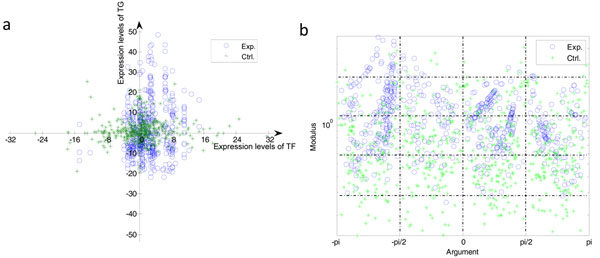
**Results of vector analyses (yeast cell cycle)** a) Distribution of sum vectors of regulation pairs based on expression level of TF and TG by vector analysis; b) Distribution of modulus and angle of expression levels vector. Y axis is log of modulus.

The vectors characterize the expression levels of one regulatory pair under each experiment condition. So it is common to calculate the sum vector of all the vectors. However, there are several different expression patterns such as correlation or inversion with or without time-shift. Sub-vectors of one regulatory gene pair in different quadrants should present different expression patterns. Therefore, sometimes sub-vectors in different quadrants may cancel out each other and result in a vain sum vector. Both the sum vector of random selected non-existing cases and that of existing cases might obtain counteractive results. After analysis of specific cases, we found that in different quadrants are distributed sub-vectors of vectors of an existing regulatory pair with perfectly synchronous expression profiles. And the sum vector of all the sub-vectors would be counteracted partly. See Figure 6a for detail.

Given the properties of time-series gene expression relationships, it is natural to calculate the sum vectors in different quadrants respectively before choosing the vector which has the largest modulus as the main representative vector of the TF-TG pair. And the sum-vector should be mapped on the modulus-argument spaces (See Figure 6b).

Compared with non-quadrant vector analysis, quadrant vector analysis can yield a much more significant classification result. Additional file 2 shows the sum vectors of regulation pairs in the experiment group coloured by correlation of the expression level (PCC). In line with the meaning of PCC, generally, main vectors in different quadrants indeed represent the different expression patterns of regulatory pairs.

#### GO Coannotation

**Figure 7:**
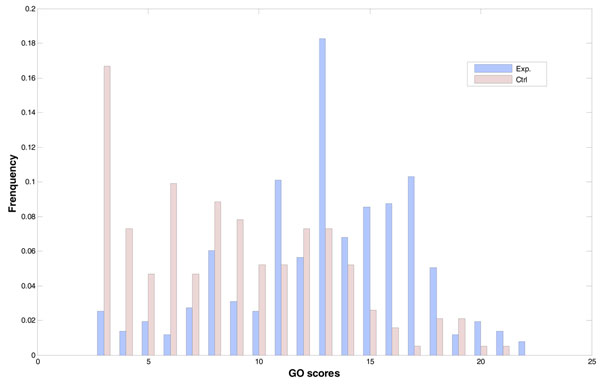
**Likelihood distributions of the GO scores of regulatory relationship expression pairs of yeast cell cycle.** The horizontal axis is grouping, and vertical axis is the frequency. Blue bars stand for experiment (case) groups with true regulatory pairs, and pink bars stand for control groups.

We took GDS2318 dataset for example and calculated the GO scores. The results are shown in Figure 7.

As we expected, the frequency of the non-existing control cases declined while GO score increased. Meanwhile, the existing gene regulatory pairs showed increasing frequencies, suggesting that gene pairs with true regulatory relationship are more likely to emerge in the same biological process.

#### Likelihood ratio

The distribution of experimental and control groups according to the GO scores are perceptibly different, and it is easy to prove the existence of differences in distribution by statistical methods. However, the underlying meaning of the distribution has been left undisclosed. The positive likelihood ratio is a good option, for it could indicate the probability of the existence of the existing regulatory relationship and be used for integration of the parameters with Bayesian model.

**Figure 8:**
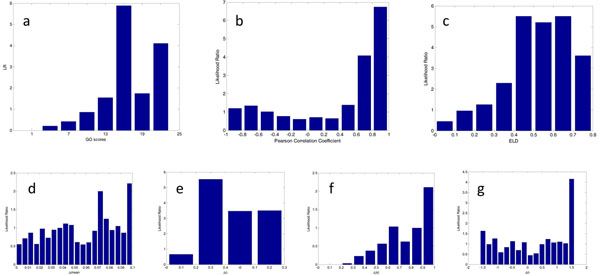
**Parameters contributing to the probability prediction (yeast cell cycle).** a) Positive likelihood ratio in different bars of GO scores; b) Likelihood ratio distribution of regulation pairs based on PCC; c) Likelihood ratio distribution of regulation pairs based on ELD; d) Likelihood distribution of the differences in mean value of expression levels; e) Likelihood distribution of the differences in standard deviation of expression levels; f) Likelihood distribution of the differences in modulus of expression levels; and g) Likelihood distribution of the differences in angle of expression levels.

First we calculated the likelihood ratio (LR), Figure 8.

As shown in Figure 8a, corresponding to the GO score, *LR*s are higher in those bars where GO scores are higher, indicating that the probability is higher when GO score is relatively high as the positive likelihood ratio represents the probability. This result corresponds with the analysis above.

### The Bayesian model integrating multiple evidences is proved to be highly efficient

#### Joint likelihood ratio

Since some parameters we introduced are paired, the respective use of their *LR*s might be unreasonable. Therefore we can combine the paired parameters to calculate the joint likelihood ratio of the joint parameter groups.

**Figure 9:**
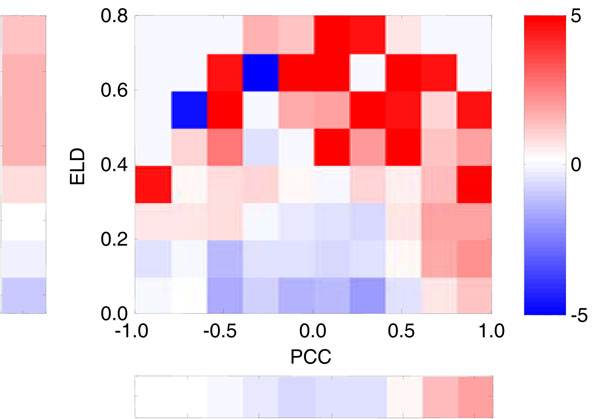
**Distribution of log likelihood ratio in PCC-ELD space (middle-right upper) compared with PCC (left bar) and ELD (bottom bar) individually (yeast cell cycle).** Red stands for positive, blue stands for negative, and the saturation shows degree

See Figure 9. Compared with Figure 5, the blue region near the origin shows low positive probability of the candidate pairs in this region. The regions with lower PCC and higher ELD are mainly coloured by red. This means that pairs located in these regions tend to be with true regulation. Given the characters of the expression levels, a possible explanation is that the existing pairs in these regions have different expression profiles. This character can be measured by the parameter ELD. The ELD of existing pairs with lower PCC is quite different from that of non-existing pairs. Compared with PCC or ELD individually, the joint likelihood ratio has a relatively strong discriminability.

Besides, calculating joint likelihood ratio of ELD and PCC can save the trouble of proving the linear independence of PCC and ELD. And this method can be used for parameters groups e.g. modulus-angel groups naturally.

#### Integration by Bayesian model

**Figure 10:**
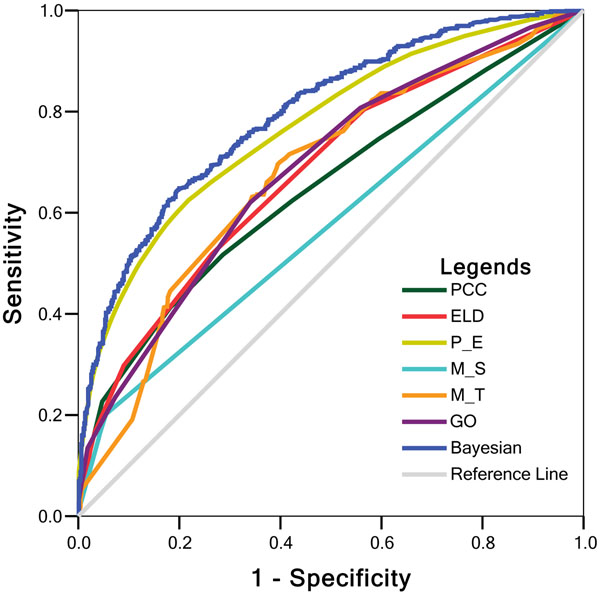
**ROC curve for parameters and integrated Bayesian model using 5-fold cross-validations against the gold standard datasets (yeast cell cycle).** P_E: PCC-ELD; M-S: Δmean-Δδ, M_T: |x|-θ.

In Figure 10, the resultant ROC curves [19] are illustrated. Each point on the ROC curve of each parameter denotes the sensitivity and specificity obtained from one test against a particular *LR*_cutoff_. The area under the curve (AUC) of ROC is an indicator of the efficacy of the individual parameter or integrated model. An ideal test with 100% sensitivity and 100% specificity has an AUC 1.0, while a non-informative prediction has an area 0.5, indicating it may be achieved randomly. The more the AUC of a test approximates 1.0, the higher the overall efficacy. We find that our improved Bayesian model has the largest AUC (0.8), which suggests it is better able to classify the true regulations against the test datasets. There is no doubt that the integrated model has the highest accuracy. For specific values of AUC, please refer to Additional file 3. When the sensitivity is set at a relatively high value 0.8, the specificity can reach up to 0.6. When the specificity is set at a relatively high value 0.8, the sensitivity can be 0.65.

### Time delay character for the prediction of the direction of regulatory relationships

Different numbers of peaks are tested respectively. Both mean and median values have been considered.

**Figure 11:**
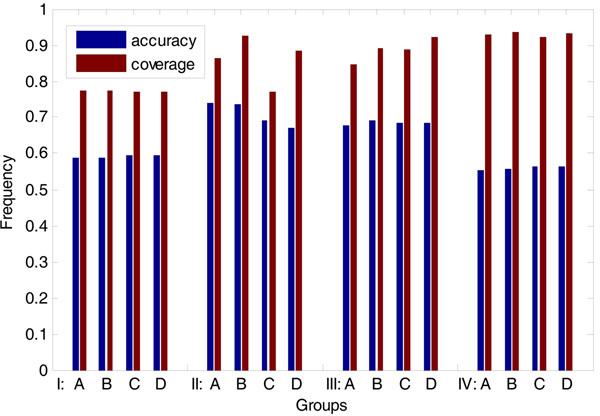
**Accuracy and coverage of predicted directions of the regulatory relationship (yeast cell cycle).** Group I, II, III and IV means 1, 2, 3 and 4 peaks of expression levels are considered. Group A and B are based on the mean value of expression levels, and group C and D based on the median value. Meanwhile, group A and C consider the existence of time delay only, and group B and D consider the amount of time-delay.

As showed in Figure 11, the condition with 2 peaks based on the amount of time-delay value is reasonable. The accuracy of the selected condition is 0.74 and the coverage is up to 0.93.

### Comparison with other methods

Typical existing methods include clustering algorithms, Bayesian networks, mutual information theory, as well as ordinary differential equations. Bansal *et al.* have compared these representative algorithms based on the simulated datasets [20].

We tested our approach on the same artificial datasets as [20], including both dynamic (time-series) and steady-state (global and local perturbation) expression dataset. Genes in the artificial network are perturbed then measured at several time-series points to construct dynamic time series microarray data. And all the genes are perturbed and measured to generate global steady-state expression data. Local means only a single gene is perturbed during the artificial experiment. The results are shown in Table 1. According to adjusting threshold *LR,* we can get result of either PPV/Se priority, or keep balance of both of them.

**Table 1:** Result of our approach on simulated dataset

Conditions	# of genes	# of samples	Direction	Se priority		Balanced		PPV priority	

				PPV	Se	PPV	Se	PPV	Se
	10	100	u	0.85	0.92	0.87	0.87	0.91	0.26
Global			d	0.41	0.89	0.51	0.78	0.71	0.63
	100	1000	u	0.20	0.95	0.30	0.28	0.99	0.05
			d	0.11	0.93	0.25	0.16	0.93	0.09

	10	10	u	-	-	0.58	0.85	-	-
Local			d	0.57	0.93	0.78	0.78	0.94	0.63
	100	100	u	0.25	0.92	0.57	0.50	0.95	0.10
			d	0.18	0.89	0.33	0.60	0.97	0.10

	10	100	u	0.47	0.97	0.67	0.79	0.93	0.63
Dynamic			d	0.49	0.96	0.64	0.67	0.92	0.48
	100	1000	u	0.21	0.90	0.42	0.23	0.55	0.10
			d	0.11	0.91	0.39	0.21	0.94	0.11

Both dynamic and steady-state (Global and local) expression dataset has been tested. Please refer to Section “Comparison with other methods” for the “conditions” description of “Global”, “Local” and “Dynamic”. PPV: positive predicted value; Se: sensitivity; u: undirected; d: directed.

Compared with algorithms tested by [20], our approach is better for dynamic (time-series) expression datasets. As shown in Table 1, in all these cases, while PPVs of our approach are equal to or slightly greater than those of the methods in [20], the corresponding Sensitivities are greater. For steady-state expression of global perturbation, our result is comparable with methods in [20]. Besides, our method performed on smaller network is somewhat better than that on the larger one. And the undirected predictions are slightly better than the directed ones.

## Discussion

In our research, besides the commonly used PCC, we proposed ELD, Δmean-Δδ, and |x|-θ as new parameters based on dynamic variation, as well as vector analysis. The parameter ELD represents the variation range character of the expression levels, and may prevent non-ideal cases from false classification by PCC alone. Then, Δmean-Δδ has an acceptable definition and accuracy. In vector analysis, we found that even the sub-vectors of a true regulatory gene pair with perfectly synchronous expression profiles are still distributed in different quadrants, and the sum vector might counteract partly. Vectors of one regulatory gene pair in different quadrants might represent different patterns. Therefore, we calculated the sum vectors in different quadrants respectively, and then chose the vector which has the largest modulus as the main representative vector of one TF-TG pair. Compared with non-quadrant vector analysis, the difference of distributions of modulus and argument is significant.

Also, we analyzed the functional co-annotation of transcription factors and their target genes, and then selected GO score as another parameter. As expected, the frequency of the non-existing control cases declined while GO score increased. Meanwhile, the existing gene regulatory pairs showed increasing frequencies, suggesting that gene pairs with true regulatory relationship have better chance of emerging in the same biological process.

Subsequently, we considered the Bayesian model for the likelihood ratio integration. Then the result was fairly acceptable. In our cases, some parameters we introduced are paired. We therefore combined the parameters to calculate the joint likelihood ratio of the joint parameter groups. The joint likelihood ratios of paired parameters make the *LR*s seems reasonable and there is no need to prove the linear independence for the parameters.

Our approach is mainly based on several novel parameters, which could be intuitive indicators. We introduced these parameters to describe characters of microarray expression data of regulating gene pairs. These features include the variation of expression level, the divergence of statistical characters, and the consistent degree of representative measurements. Additionally, our approach is much less costly than some mainstream methods. Therefore, our approach can serve as a fast pre-process strategy for microarray data analysis.

Some papers argue that inferring regulatory relationship based on microarray has inherent faults. First, the similarity of the expression profile suggests nothing more than a statistical dependency between two genes, not a direct causal relationship. The verification of the relationship requires other evidences, such as ChIP-chip data, Y2H or other wet experiments. Second, essential genes [21] which are always expressed in the cell cannot be disturbed by knockdown or knockout. Therefore microarray experiments do not work well on these essential genes. Third, microarray is a kind of high-throughput analysis technology after all, so it cannot be very precise. Genes with a slim expression level can hardly be detected accurately.

Recently a series of reports indicates that the microarray might be replaced by fast high-throughput sequencing [22,23], which, however, cannot be made as inexpensive and efficient as microarray now. In the future, microarray might be used to meet more specific research needs, such as fast elementary filter or test. Therefore complex models might not be suited to the fast measurement of the microarray. Though our approach is more or less rough and far from perfect, we still believe some simple indicators based on uncomplicated characters would reveal complex behaviour.

## Conclusions

With the rapid deposition of the microarray data in recent years, microarray data have become an increasingly important data source for bioinformatics research. On the basis of microarray data, constructing gene regulatory networks has also become a hotspot. By constructing the gene regulatory network, we can identify the complicated regulatory relationships, uncover the regulatory patterns in the cell, and gain the global view of the biological process. In this paper, we present some novel parameters to uncover potential characters of regulatory relationships. In addition to routine description of the similarity of the expression levels, our proposed parameters measured range of the variation and the statistic feature of expression levels, consistency of sub-vectors of the expression level, as well as functional co-annotation of regulating pairs. Unlike other global expression profiles computational methods, our approach is mainly based on several novel parameters, which could be intuitive indicators. And our parametric approach can serve as a fast approach for regulatory relationship mining.

## Materials and methods

### Datasets

As a simple but important organism, yeast *Saccharomyces cerevisiae* is a proper target of research. First we set up an experiment group of regulatory element pairs with existing (true) regulatory relationship. These existing pairs were obtained from published literature [24,25]. In addition, we constructed a control group for training dataset. The pairs in control group were randomly selected and known existing regulatory pairs had been excluded. During the research, we observed that the ratio of existing and non-existing pairs in the training set would affect the result. The increases of negative data in training set induce a decrease of positive prediction value with the fixed sensitivity. It indicates that suitable ratio of positive and negative must be noticed. The result is shown in Additional file 4. Time series datasets derived from cell cycle experiments were downloaded from GEO dataset in PubMed. The GO annotations are retrieved by GOfact [26].For a proper comparison with other methods, artificial datasets is an appropriate choice. *In silico* data could control the noise levels of the data. Here, we used the datasets in [20], which was generated an artificial dataset by linear ODEs, with the mean of white noise 0 and standard deviation 0.1.

### Bayesian model

Bayesian model has been widely used for integrating proofs [27,28]. Likelihood ratio is the probability of observing an existing gene pair in predictive datasets divided by the probability of observing the non-existing gene pair in predictive datasets [29]. Here prior odds are the chance of choosing a pair of regulatory genes from all candidate gene pairs.

Posterior odds of regulation is the product of prior odds and likelihood ratio.

The prior odds of regulation are the probability of occurrence of the positive divided by the probability of occurrence of the negative.

In other words, *P*(*positive*) is the probability of finding a pair of genes in all the possible regulation, and the *P*(*negative*) is the probability of finding a non-regulation pair in all the possible regulation. The posterior odds are often decided by the mean numbers of regulation in all the known regulation. So the posterior odds are

*f*_i_ means the number of gene pairs in the dataset i. And the Bayesian method is considered

*O_post_* = *O_prior_* × *LR*(*f*_1_…*f_n_*)

Subsequently,

In this formula, *LR* means likelihood ratio, and *positive* means gold standard positive dataset of gene pairs where real regulatory relationship exists. And *negative* is the gold standard negative dataset in which no gene pair has any regulation.

Finally, under certain assumptions, such as the predicted dataset, individual or non-redundant, the likelihood can be counted by the product of likelihoods of individual sets.

This is also known as the naïve Bayesian network.

## List of abbreviations

AUC: area under the curve; ELD: expression level differences; GEO: Gene Expression Omnibus; GO: Gene Ontology; LR: likelihood ratio; ODE: ordinary differential equation; PCC: Pearson correlation coefficient; PPV: positive predictive value; ROC: receiver operating characteristic; Se: sensitivity; TF: transcriptional factor; TG: target gene

## Competing interests

The authors declare that they have no competing interests.

## Authors' contributions

W. Liu and D. Li designed and implemented the whole methodology and the computation framework. Q. Liu provided constructive discussions and refinement of the formula. Y. Zhu monitored the whole framework and revised the manuscripts. F. He directed the whole project and revised the manuscript. All the authors have read and agreed to the manuscript.

## Supplementary Material

Additional file 1 **Left:** experience group. A) An ideal expression profile of coregulatory pair with a relatively higher |PCC|. C) An expression profile of a true regulatory pair with a lower |PCC|. **Right:** control group. B) A typical expression profile of a non-existing pair with a lower |PCC|. D) A non-existing pair whose variation ranges of expression levels are relatively smaller, reduced a relatively higher |PCC|. Click here for file

Additional file 2 E.L.: expression level. Red stands for positive correlation, blue stands for negative correlation, and the saturation shows the correlation degree. Sum vectors of regulation pairs in experiment group were coloured by PCC. Compared with the meaning of PCC, generally, main vectors in different quadrants indeed represent the different expression patterns of regulatory pairs. Click here for file

Additional file 3 Our proposed model has a relative large AUC (0.8), which suggests it is able to predict regulations efficiently. Click here for file

Additional file 4 PPV1 to PPV7 in scales stand for positive and negative ratio in training set is 1:1 to 1:7; and the scale “PPV” stands for all the pairwised cases in the network composed by all the genes have been considered. Click here for file
