# Supplementary figures and images for: A novel parametric approach to mine gene regulatory relationship from microarray datasets
Source: BMC Bioinformatics. 2010 Dec 14;11(Suppl 11):S15. doi: 10.1186/1471-2105-11-S11-S15 (PMC3024862; doi:10.1186/1471-2105-11-S11-S15)

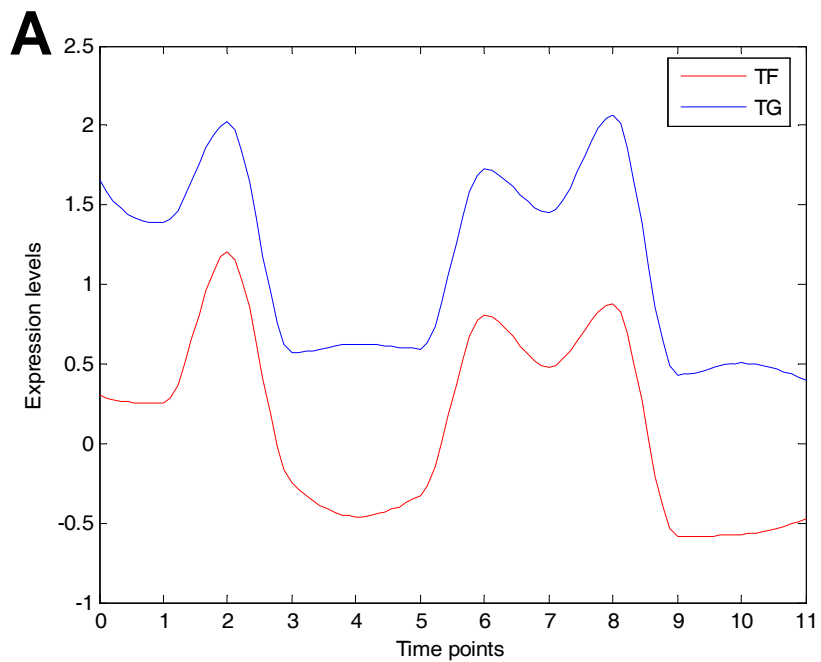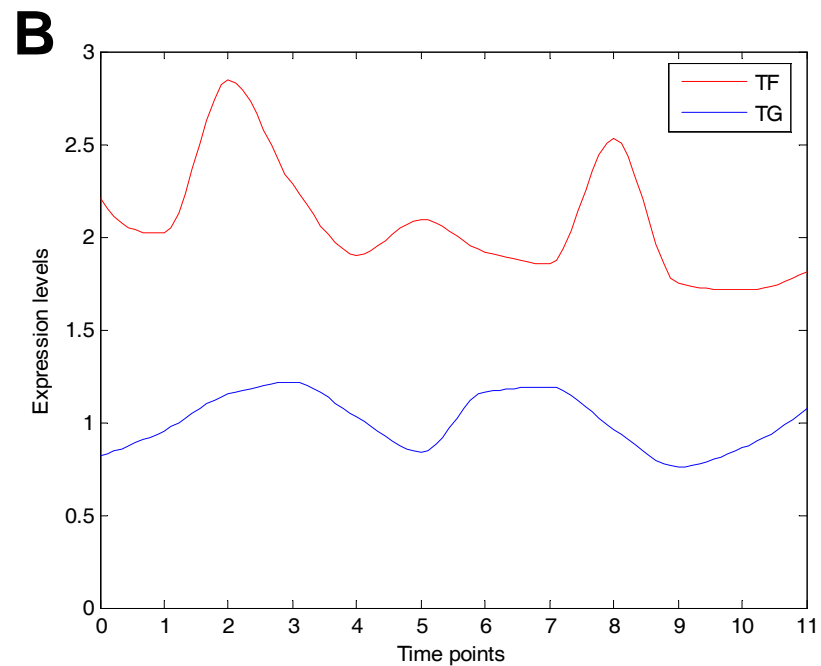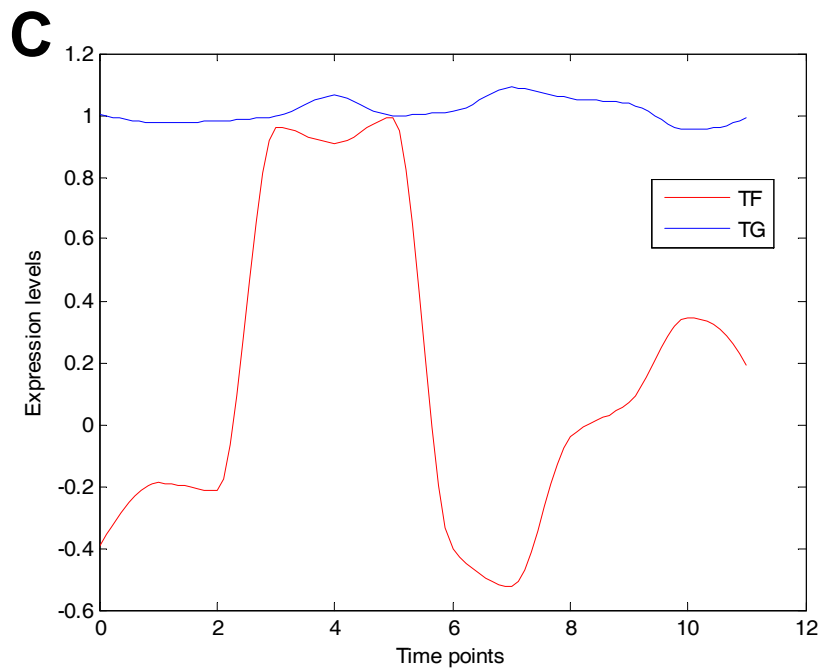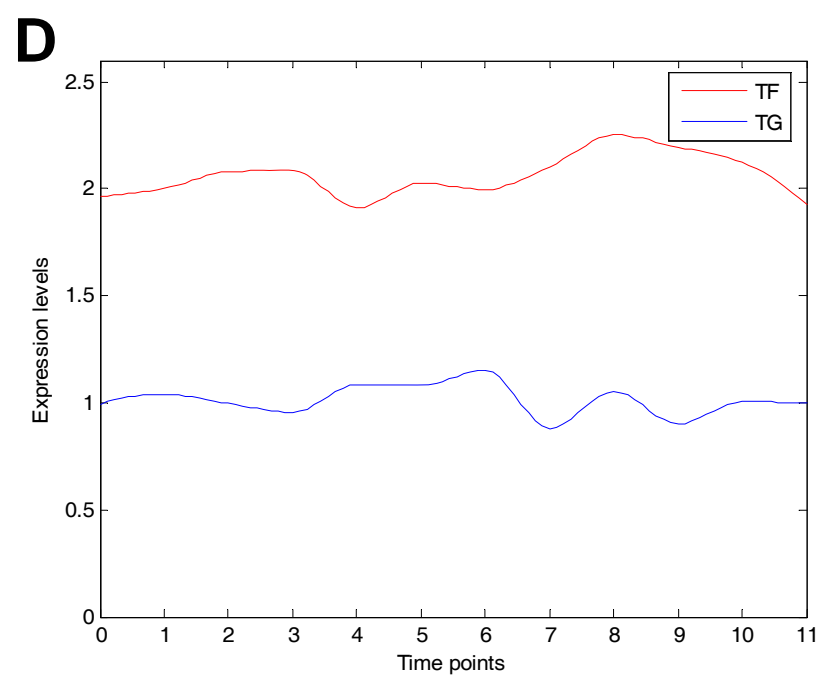

Supplement: Additional file 1 — Left: experience group. A) An ideal expression profile of coregulatory pair with a relatively higher |PCC|. C) An expression profile of a true regulatory pair with a lower |PCC|. Right: control group. B) A typical expression profile of a non-existing pair with a lower |PCC|. D) A non-existing pair whose variation ranges of expression levels are relatively smaller, reduced a relatively higher |PCC|. [file 1471-2105-11-S11-S15-S1.pdf]

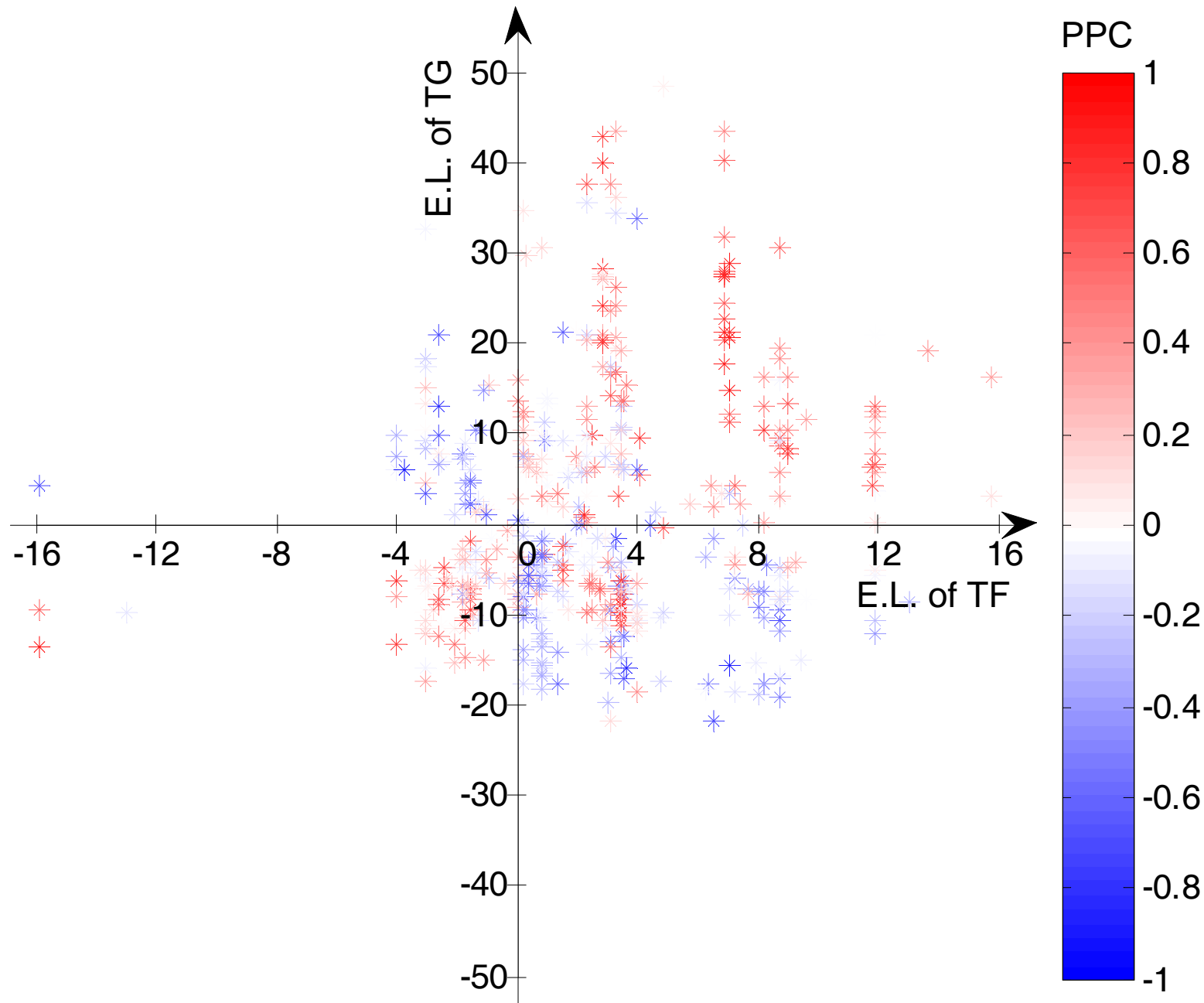

Supplement: Additional file 2 — E.L.: expression level. Red stands for positive correlation, blue stands for negative correlation, and the saturation shows the correlation degree. Sum vectors of regulation pairs in experiment group were coloured by PCC. Compared with the meaning of PCC, generally, main vectors in different quadrants indeed represent the different expression patterns of regulatory pairs. [file 1471-2105-11-S11-S15-S2.pdf]

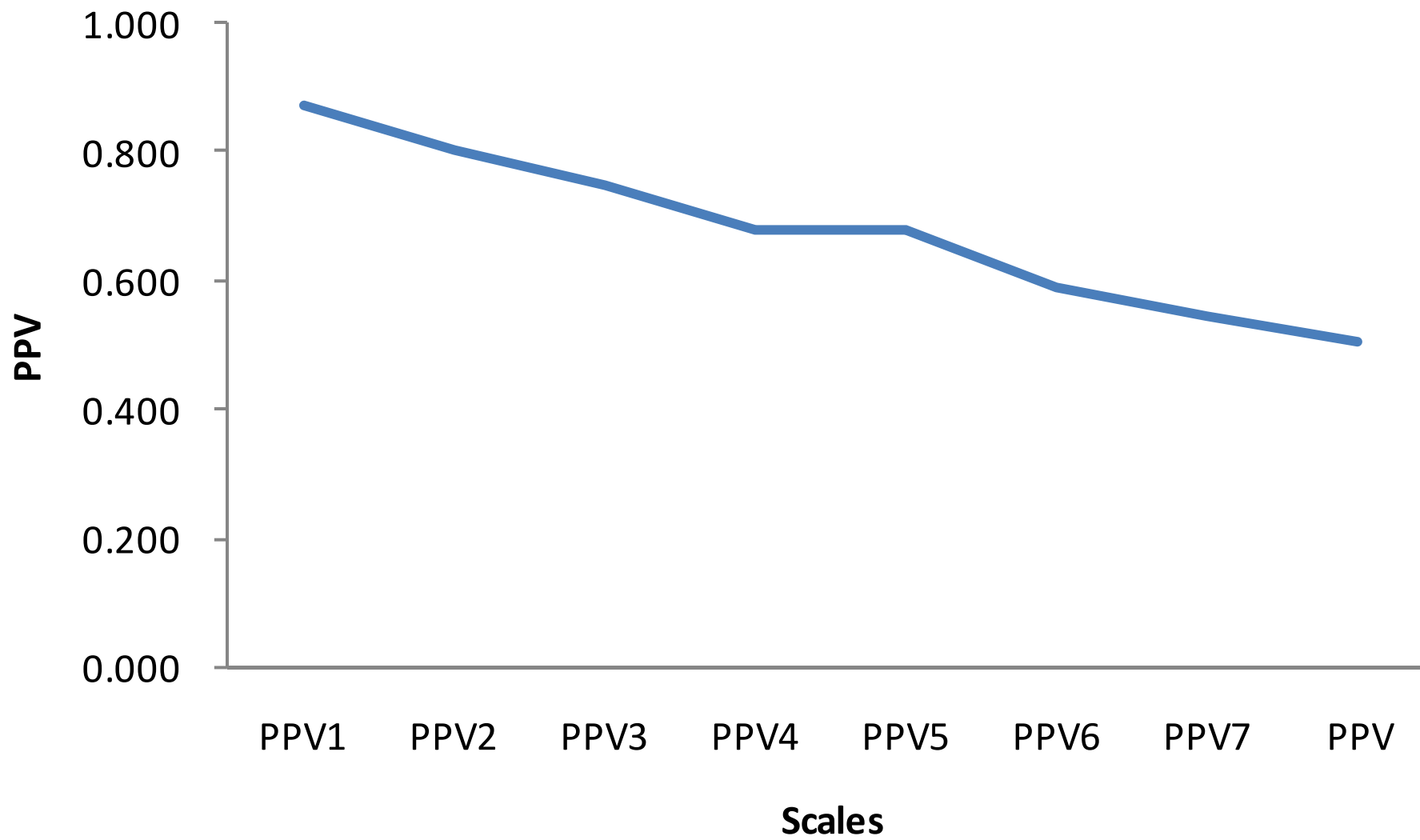

Supplement: Additional file 4 — PPV1 to PPV7 in scales stand for positive and negative ratio in training set is 1:1 to 1:7; and the scale “PPV” stands for all the pairwised cases in the network composed by all the genes have been considered. [file 1471-2105-11-S11-S15-S4.pdf]
