# Supplementary material for: A novel parametric approach to mine gene regulatory relationship from microarray datasets
Source: BMC Bioinformatics. 2010 Dec 14;11(Suppl 11):S15. doi: 10.1186/1471-2105-11-S11-S15 (PMC3024862; doi:10.1186/1471-2105-11-S11-S15)
Supplement: Additional file 3 — Our proposed model has a relative large AUC (0.8), which suggests it is able to predict regulations efficiently. [file 1471-2105-11-S11-S15-S3.pdf]

Additional file 3 - Area under the curve of ROC.

Our proposed model has a relative large AUC (0.8), which suggests it is able to predict regulations efficiently.

P_E: PCC-ELD; M_S: Δmean-Δδ; M_T: |x|-θ.

Parameters	AUC
PCC	0.65
ELD	0.68
P_E	0.77
M_S	0.57
M_T	0.68
GO	0.68
**Bayesian**	**0.80**
